# Pancreatic-Type Acinar Cell Carcinoma of the Duodenum: A Case Report and Literature Review

**DOI:** 10.70352/scrj.cr.25-0583

**Published:** 2025-12-16

**Authors:** Daisuke Shirai, Seiko Hirono, Masaharu Tada, Kenjiro Iida, Hideaki Sueoka, Ami Kurimoto, Mizuki Yoshida, Kazu Harada, Chisato Ohe, Hiroshi Kono, Ikuo Nakamura

**Affiliations:** 1 Division of Hepato-Biliary-Pancreatic Surgery, Department of Gastroenterological Surgery, Hyogo Medical University, Nishinomiya, Hyogo, Japan; 2 Bellland General Hospital, Sakai, Osaka, Japan; 3 Department of Diagnostic Pathology, Hyogo Medical University, Nishinomiya, Hyogo, Japan

**Keywords:** acinar cell carcinoma, ectopic pancreas, duodenum

## Abstract

**INTRODUCTION:**

Acinar cell carcinoma (ACC) is a rare malignant tumor arising from pancreatic exocrine cells. While it typically originates in the pancreas, ectopic occurrences, especially in the duodenum, are extremely uncommon. Few reports exist of duodenal ACC, particularly those presenting as large tumors.

**CASE PRESENTATION:**

An 83-year-old man presented with melena. Endoscopy and imaging revealed a large mass, approximately 7 cm in size, extending from the duodenal bulb to the descending portion of the duodenum. Contrast-enhanced CT showed no evidence of extramural invasion or distant metastasis. Although biopsy confirmed malignancy, a definitive diagnosis could not be established preoperatively. The patient underwent pancreaticoduodenectomy. Postoperative histopathological examination, including immunohistochemical staining positive for trypsin, confirmed the diagnosis of pancreatic-type ACC, likely arising from ectopic pancreatic tissue in the duodenum.

**CONCLUSIONS:**

We report a rare case of a giant pancreatic-type ACC of the duodenum, which was strongly suspected to have arisen from ectopic pancreatic tissue. This case highlights the diagnostic challenges associated with pancreatic-type ACC of the duodenum and adds valuable information to the limited literature on this rare entity.

## Abbreviations

ACC
: acinar cell carcinoma
FDG
: fluorodeoxyglucose
ISGPF
: International Study Group on Pancreatic Fistula
PSA
: prostate-specific antigen
SUVmax
: maximum standardized uptake value

## INTRODUCTION

ACC is a rare malignancy, accounting for 1%–4% of pancreatic tumors, and preoperative diagnosis is difficult due to nonspecific imaging, often requiring histopathology for confirmation.^1,2)^ ACC generally has a better prognosis than pancreatic ductal adenocarcinoma, with 5-year survival rates of 21%–40%.^1–3)^

Ectopic pancreas is a congenital anomaly characterized by the presence of pancreatic tissue lacking anatomical or vascular continuity with the main pancreas. It is most commonly located in the stomach, duodenum, and proximal jejunum, accounting for the majority of reported cases, and its prevalence has been estimated at approximately 0.5%–13.7% in autopsy series, with most lesions remaining asymptomatic and detected incidentally.^4)^

ACC arising from ectopic pancreatic tissue is even more uncommon. While a few cases of ACC originating in the stomach or liver have been reported, occurrences in the duodenum are extremely rare. Here, we report a case of a giant ACC arising in the duodenum that was successfully resected, along with a review of the literature.

## CASE PRESENTATION

**Fig. 1:**
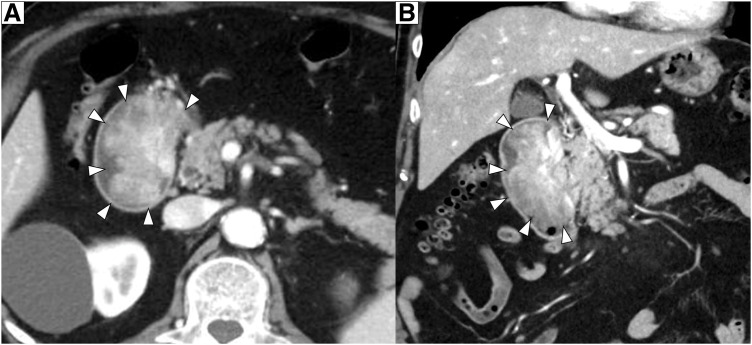
Contrast-enhanced abdominal CT. (**A**) Axial. (**B**) Coronal. A 73-mm mass lesion extending from the duodenal bulb to the descending portion of the duodenum was observed (white triangles). A tumor protruding into the duodenal lumen was observed, without clear evidence of extramural invasion.

**Fig. 2:**
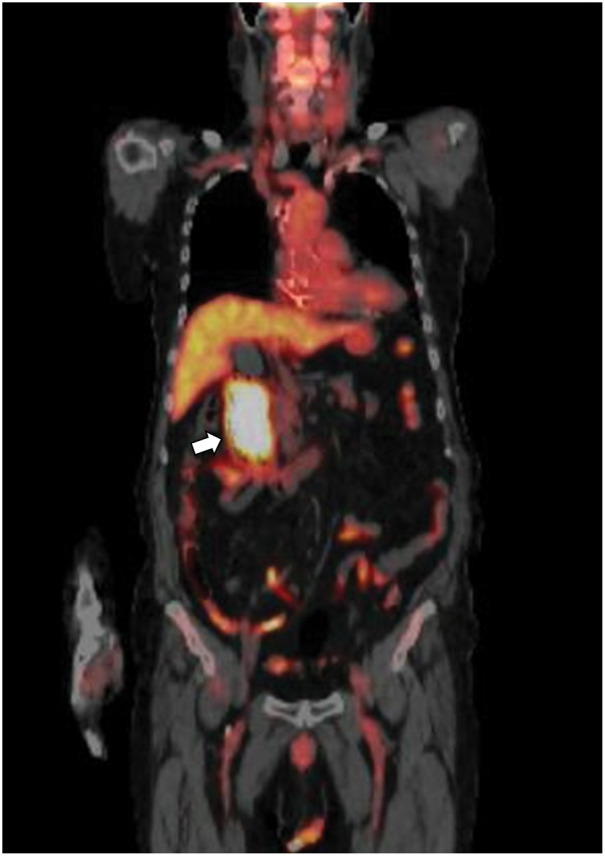
PET-CT. The mass lesion demonstrated abnormal FDG uptake with a maximum standardized uptake value of 6.57. FDG, fluorodeoxyglucose

**Fig. 3:**
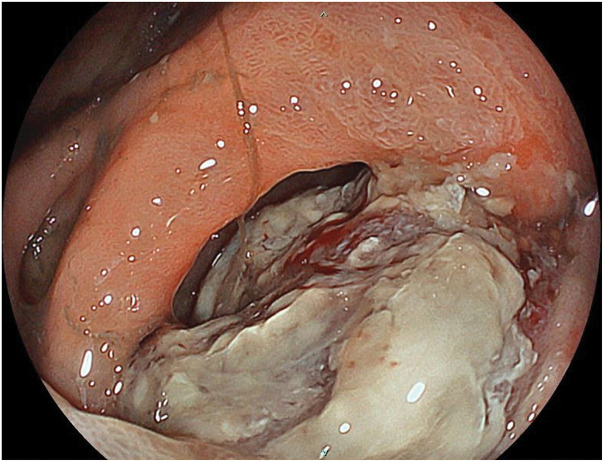
Upper gastrointestinal endoscopy. A protruding lesion was extending from the pyloric ring to the region just above the major papilla. Endoscopy was able to pass through the tumor.

An 83-year-old male presented with a chief complaint of melena. His past medical history included prostate cancer, for which he underwent prostatectomy and radiation therapy in 2015 (pT3aN0M0, Stage III); atrioventricular block, for which a pacemaker had been implanted; and hypertension and dyslipidemia managed with oral medications. There was no notable family history. The patient visited our hospital’s department of general internal medicine with a chief complaint of melena. Upper gastrointestinal endoscopy revealed a large mass-like lesion extending from the duodenal bulb to the descending portion of the duodenum. He was referred to our department for surgical treatment. His height was 167 cm, and his weight was 78 kg. Physical examination revealed a flat and soft abdomen without tenderness, and no palpable mass was detected. Laboratory tests, including a hemoglobin level of 9.6 g/dL (indicating anemia), showed no significant abnormalities in liver and biliary enzymes or pancreatic enzymes. Tumor markers were also within the normal range, with a carcinoembryonic antigen level of 1.5 ng/mL and a carbohydrate antigen19-9 level of 11 U/mL. Contrast-enhanced abdominal CT showed a 73-mm mass lesion extending from the duodenal bulb to the descending portion of the duodenum (**Fig. 1**). There was no apparent extramural extension, and although the tumor was adjacent to the pancreas and bile duct, no clear evidence of direct invasion was observed. On PET-CT, the mass lesion demonstrated abnormal FDG uptake with a SUVmax of 6.57, but no abnormal uptake suggesting distant metastasis was identified (**Fig. 2**). Upper gastrointestinal endoscopy demonstrated a protruding lesion extending from the pyloric ring to the region just above the major papilla, suggestive of duodenal cancer (**Fig. 3**). Histopathological examination of the biopsy specimen showed pleomorphic tumor cells proliferating in alveolar and sheet-like patterns. Immunohistochemistry revealed positivity for AE1/AE3, weak positivity for NKX3.1, and negativity for PSA, raising the possibility of metastasis from previously treated prostate cancer, though a definitive conclusion could not be made.

Based on these findings, a diagnosis of either primary duodenal carcinoma or duodenal metastasis from prostate cancer was considered. Given the tumor’s large size, staging laparoscopy was performed to assess for peritoneal dissemination. During laparoscopy, no liver metastasis or peritoneal dissemination was found. A large tumor was visualized within the duodenum, but there was no evidence of serosal surface exposure. Peritoneal lavage cytology was negative for malignant cells. Accordingly, an open pancreaticoduodenectomy was subsequently performed on a different day. The tumor was located within the duodenum and presented as a soft, elastic mass, with no evidence of invasion into adjacent organs. The stomach was transected approximately 5 cm proximal to the pyloric ring, at a safe distance from the tumor, and intraoperative frozen section analysis of the gastric stump showed no evidence of malignancy. Regional lymphadenectomy was performed, and reconstruction was carried out using a modified Child method. The operative time was 6 h and 24 min, with a blood loss of 365 mL.

**Fig. 4:**
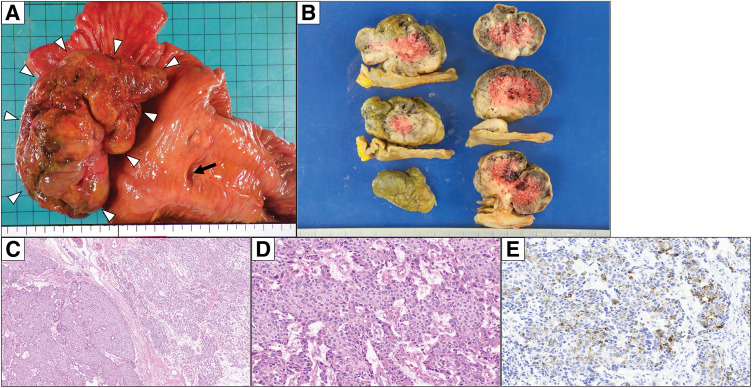
Histopathological and immunohistochemical examinations of the resected specimen. (**A**) Macroscopic findings. An approximately 100-mm pedunculated tumor arising from the duodenum (white triangles). Black arrow: Ampulla of Vater. (**B**) Tumor cut surface after formalin fixation. (**C**) Hematoxylin and eosin stain (×40). The tumor cells exhibited eosinophilic granular cytoplasm and nuclear pleomorphism, with some areas showing eosinophilic granules resembling pancreatic acinar cell zymogen granules. (**D**) Hematoxylin and eosin stain (×200). (**E**) Trypsin positive (×200).

The resected specimen revealed a pedunculated tumor approximately 100 mm in size arising from the duodenum (**Fig. 4A** and **4B**). The surgical margins were negative for tumor involvement, and no lymph node metastasis was identified (0/23). Histologically, the tumor was mainly located in the lamina propria and exhibited glandular, cribriform, and acinar growth patterns. The tumor cells had eosinophilic granular cytoplasm with nuclear pleomorphism, and some areas contained eosinophilic granules resembling zymogen granules of pancreatic acinar cells on hematoxylin and eosin staining (**Fig. 4C** and **4D**). Immunohistochemically, the tumor was partially positive for trypsin (**Fig. 4E**). Notably, in both the preoperative biopsy and the resected specimen, immunostaining for NKX3.1 and PSA was performed, revealing partial NKX3.1 positivity and PSA negativity. Based on the acinar morphology, eosinophilic cytoplasm, and trypsin positivity, metastatic prostate cancer was excluded. These findings supported a pathological diagnosis of pancreatic-type ACC of the duodenum. Because the tumor was centered in the lamina propria of the duodenal wall, a tumor arising from ectopic pancreatic tissue was strongly suspected; however, non-neoplastic pancreatic tissue contiguous with the lesion was not identified due to the tumor’s large size, precluding a definitive confirmation of ectopic pancreatic origin.

Postoperatively, the patient developed a Grade B pancreatic fistula according to the ISGPF classification^5)^ that required drain replacement without any symptoms and was discharged on POD 47. No adjuvant chemotherapy was administered, and the patient remains recurrence-free at 6 months postoperatively under close observation.

## DISCUSSION

ACC is a rare malignant tumor originating from pancreatic exocrine acinar cells and accounts for only 1%–2% of all pancreatic neoplasms.^3)^ Extra-pancreatic ACC is extremely rare, with only a few case reports available. Among the 15 reported cases of gastrointestinal ACC,^6–19)^ 10 arose from the stomach, and only 2 from the duodenum, and the average tumor diameter in reported cases is 4.8 cm (**Table 1**). The duodenal tumor of the present case measured 10 cm, making it the largest reported to date. According to the 15 reports, only 1 case was diagnosed preoperatively, highlighting the difficulty of diagnosis before surgery due to the rarity of this disease.

**Table 1:** Reported cases of acinar cell carcinoma arising from the gastrointestinal tract

Case	Year	Author	Age	Sex	Tumor location	Tumor size (cm)	Preoperative diagnosis	Treatment	Outcome
1	2004	Sun	86	M	Stomach	5.0	PDA	Partial gastrectomy	NM
2	2007	Mizuno	73	M	Pylorus	7.0	GIST, ML	PD	Alive (11 months)
3	2007	Kawakami	65	F	Vater	1.2	Carcinoma	PD	Alive (19 months)
4	2009	Chiaravalli	65	F	Colon	4.0	NM	Colonic segment resection	Death (24 months)
5	2009	Ambrosini-Spaltro	52	M	Stomach	4.0	PDA	Subtotal gastrectomy	NM
6	2012	Coyne	77	F	Stomach	4.5	PDA	Partial gastrectomy	NM
7	2013	Hamidian Jahromi	58	M	Duodenum	2.7	NM	Duodenal resection	Alive (18 months)
8	2016	Yonenaga	67	M	Stomach	8.5	NM	Distal gastrectomy	Alive (21 months)
9	2016	Yonenaga	63	M	Stomach	6.5	PDA	Chemotherapy	Death (5 months)
10	2017	Kim	54	M	Stomach	2.7	GIST, ML	Partial gastrectomy	Alive (33 months)
11	2017	Takagi	78	F	Jejunum	8.5	PDA	Partial resection and chemotherapy	Alive (10 months)
12	2021	Uno	68	M	Stomach	1.7	PDA	ESD	NM
13	2022	Paseiro-Crespo	51	F	Stomach	8.0	Neuroendocrine tumor	Total gastrectomy	Alive (5 months)
14	2023	Chen	58	F	Stomach	1.2	PDA	Partial gastrectomy	Alive (6 months)
15	2024	Narita	61	F	Duodenum (accessory papilla)	1.0	ACC	PD	Alive (6 months)
16	2024	Our case	83	M	Duodenum	10.0	Carcinoma	PD	Alive (6 months)

ACC, acinar cell carcinoma; ESD, endoscopic submucosal dissection; F, female; GIST, gastrointestinal stromal tumor; M, male; ML, malignant lymphoma; NM, not mentioned; PD, pancreaticoduodenectomy; PDA, poorly differentiated adenocarcinoma

Ectopic pancreas is defined as pancreatic tissue located outside the normal pancreas without anatomical or vascular continuity, often found within the gastrointestinal wall, particularly in the submucosa. Reported incidence varies, but it has been found in 0.55%–13.7% of autopsy cases.^4)^ The most common site is the stomach, followed by the duodenum, which accounts for approximately 30% of all ectopic pancreas cases. It is usually asymptomatic and often found incidentally during endoscopy or surgery. Malignant transformation is rare, with a reported rate of 0.7%–1.8%.^20)^

Järvi and Laurén proposed 3 criteria for diagnosing malignancy arising from ectopic pancreatic tissue: 1) the tumor is located within or near ectopic pancreas, 2) there is a transition between pancreatic tissue and carcinoma, and 3) non-neoplastic pancreatic tissue includes acini, ducts, and islets of Langerhans.^21)^ In this case, no ectopic pancreatic tissue was found near the tumor, as suggested by Järvi and Laurén. This may be attributed to the exceptionally large size of the tumor, which likely resulted in complete replacement of the ectopic pancreatic tissue by cancer cells. Furthermore, the tumor was pathologically found to have originated from the submucosal layer of the duodenum, a characteristic feature of tumors arising from ectopic pancreas.^4)^ Based on these findings, this case was diagnosed as pancreatic-type ACC of the duodenum, likely arising from ectopic pancreatic tissue.

ACC is difficult to diagnose preoperatively due to its rarity and histological similarities to adenocarcinomas and neuroendocrine tumors. Imaging of pancreatic ACC often shows a well-circumscribed, round, hypovascular mass with a capsule.^22,23)^ It tends to grow expansively, and obstructive symptoms are uncommon. In our case, despite the large tumor occupying the duodenum, no obstructive symptoms were observed—consistent with typical ACC features. Histological diagnosis requires immunohistochemical staining for pancreatic enzymes such as trypsin, chymotrypsin, and lipase.^6)^ Markers like α1-antitrypsin and BCL10 are also useful. In this case, because the patient had a history of prostate cancer, metastatic prostate cancer was initially suspected. Immunostaining for ACC was not performed on biopsy specimens, and ACC could not be diagnosed preoperatively. As preoperative biopsy often fails to establish a diagnosis, it is important to consider ACC in the differential diagnosis based on imaging and clinical features.

We performed pancreaticoduodenectomy with regional lymph node dissection, and pathological findings showed no residual malignancy at all resection margins and no lymph node metastasis. There is no established treatment protocol due to the rarity of the disease. A few reports described that the prognosis of pancreatic ACC is more favorable than that of pancreatic ductal adenocarcinoma, with a 5-year survival rate of 21%–40%.^1–3)^ However, recurrence after surgical resection is relatively common, occurring in approximately 40%–60% of patients, with the liver being the most frequent site, followed by regional lymph nodes and the peritoneum.^1,2)^ Management of recurrent disease is not standardized due to the rarity of ACC, but some reports suggest that surgical resection and systemic chemotherapy may be considered, with FOLFIRINOX often eliciting favorable responses.^2)^ As evidence supporting the efficacy of adjuvant therapy for ACC arising from ectopic pancreas is limited, no postoperative adjuvant treatment was administered in this case. The patient remains disease-free 6 months after surgery. However, continued surveillance for recurrence is warranted.

## CONCLUSIONS

This case represents an extremely rare entity: pancreatic-type ACC likely arising from ectopic pancreas in the duodenum. Although preoperative diagnosis is challenging, features such as large tumor size and the absence of obstructive symptoms may be suggestive. It is important to consider ectopic pancreas and its potential for malignant transformation in the differential diagnosis.

## DECLARATIONS

### Availability of data and materials

The dataset supporting the conclusions of this article is included within the article.
